# Complete Description of the Three Pathways of the Complement System in a Series of 430 Patients with Rheumatoid Arthritis

**DOI:** 10.3390/ijms25158360

**Published:** 2024-07-31

**Authors:** Dara Rodríguez-González, María García-González, Fuensanta Gómez-Bernal, Juan C. Quevedo-Abeledo, Agustín F. González-Rivero, Yolanda Fernández-Cladera, Elena González-López, J. Gonzalo Ocejo-Vinyals, Alejandro Jiménez-Sosa, Beatriz González-Toledo, Miguel Á. González-Gay, Iván Ferraz-Amaro

**Affiliations:** 1 Division of Central Laboratory, Hospital Universitario de Canarias, 38320 Santa Cruz de Tenerife, Spain; dararg1@gmail.com (D.R.-G.); fuensanta95@gmail.com (F.G.-B.); afgonriv@gmail.com (A.F.G.-R.); yolanda.fernandezcladera@gmail.com (Y.F.-C.); 2 Division of Rheumatology, Hospital Universitario de Canarias, 38320 Santa Cruz de Tenerife, Spain; margagon23@hotmail.com; 3 Division of Rheumatology, Hospital Doctor Negrín, 35010 Las Palmas de Gran Canaria, Spain; quevedojcarlos@yahoo.es; 4 Division of Immunology, Hospital Universitario Marqués de Valdecilla, Instituto de Investigación Valdecilla (IDIVAL), 39011 Santander, Spain; elenagonzalez_lopez@hotmail.com (E.G.-L.); javiergonzalo.ocejo@scsalud.es (J.G.O.-V.); 5 Research Unit, Hospital Universitario de Canarias, 38320 Santa Cruz de Tenerife, Spain; ajimenezsosa@gmail.com; 6 Fundación Jimenez Díaz School of Nursing, Autonomous University of Madrid, 28040 Madrid, Spain; beatriz.gonzalez.toledo.93@gmail.com; 7 Health Research Institute, Fundación Jiménez Díaz, 28040 Madrid, Spain; 8 Division of Rheumatology, IIS-Fundación Jiménez Díaz, 28040 Madrid, Spain; 9 Deparment of Internal Medicine, University of Cantabria, 39005 Santander, Spain; 10 Department of Internal Medicine, University of La Laguna (ULL), 38200 Santa Cruz de Tenerife, Spain

**Keywords:** rheumatoid arthritis, complement system, complement pathways, complement activity assays, disease activity, rheumatoid factor, anti-citrullinated protein autoantibodies, inflammation

## Abstract

The complement (C) system is implicated in the etiopathogenesis of rheumatoid arthritis (RA). However, there is a lack of studies characterizing all three C pathways in RA patients. This study aimed to evaluate the association between an in-depth examination of the C system and RA patient characteristics, focusing on disease activity and the presence of rheumatoid factor and anti-citrullinated protein autoantibodies (ACPA). In a cohort of 430 RA patients, functional assays of the three C pathways (classical, alternative, and lectin) and serum levels of their components were assessed. Components included C1q (classical); factor D and properdin (alternative); lectin (lectin); C1-inhibitor; C2, C4, and C4b (classical and lectin); C3, C3a, and C4b (common); and C5, C5a, and C9 (terminal). A multivariable linear regression analysis showed significant positive correlations between C-reactive protein and C system proteins and functional assays, especially in the terminal and common pathways. Disease activity, measured by scores with or without acute phase reactants, positively correlated with the classical pathway functional test and terminal pathway products. Conversely, rheumatoid factor or ACPA presence was associated with lower classical pathway values and decreased C3a and C4b levels, suggesting complement depletion. In conclusion, RA disease activity increases C molecules and functional complement assays, while rheumatoid factor or ACPA positivity is linked to C consumption. Our study offers a detailed analysis of the complement system’s role in RA, potentially guiding the development of more targeted and effective treatment strategies.

## 1. Introduction

The complement (C) system plays a crucial role in the innate immune system, operating in synergy with antibody-triggered reactions. This collaboration is reflected in its name, “complement” [1]. This system comprises around 60 proteins found in serum and cell membranes. The C system is important in the defense of the host against microbes, particularly bacteria. It also serves as a mechanism to identify and clear injured tissue and cellular debris. Thus, it is a major player in innate immunity and an effector arm of the humoral immune system [2]. Proteins of the C are organized into three separate yet interconnected activation pathways: the classical, alternative, and lectin cascades. Additionally, there exist a shared terminal lytic pathway and a complex web of regulators and receptors [3].

Each C cascade is triggered in a distinct manner, yet all lead to the activation of C3 and its deposition on a target such as C3b, which is the major goal of C. The classical pathway is triggered by antibodies. It becomes engaged when immunoglobulin M (IgM) or immunoglobulin G (IgG) antibodies bind to antigens (such as viruses, bacteria, or autoantigens). The lectin pathway is specialized for the prompt recognition of repetitive carbohydrate patterns on the surface of microbial pathogen targets. The alternative pathway is an ancient surveillance system and represents the original extracellular C system. It does not require the presence of antibodies or lectins to become activated. It is continuously turning over (so called “tick-over”) at a low level due to the presence of a labile thioester bond in C3. The C cascade needs to be tightly regulated to avoid overactivation and inflammatory pathologies. Inadequately controlled C activation may underlie the pathogenesis of several processes like infection; cancer; renal diseases; and immunomodulated processes like systemic lupus erythematosus, antiphospholipid syndrome, cryoglobulinemia, anti-neutrophil cytoplasmic antibody-associated vasculitis, autoimmune hemolytic anemia, or myasthenia gravis [4].

Rheumatoid arthritis (RA) is the most common chronic form of inflammatory arthritis, affecting approximately 1 percent of the population. It results from complex interactions between genes and the environment, leading to a breakdown of immune tolerance and to synovial inflammation in a characteristic symmetric pattern. Distinct mechanisms promote and regulate inflammation and matrix destruction, including damage to bone and cartilage [5]. The classification of RA patients can be further refined based on their serostatus, specifically the presence or absence of rheumatoid factor and anti-citrullinated protein antibodies (ACPA). These serological markers not only aid in diagnosing RA but also provide insights into the disease’s pathophysiological mechanisms and prognostic implications. Their presence is associated with a more aggressive disease and is believed to play a direct role in pathogenesis by forming immune complexes that drive inflammation. Consequently, seropositive RA typically presents with more severe clinical manifestations, including greater joint damage and extra-articular features such as nodules, lung involvement, and vasculitis.

Inadequately controlled C activation has emerged as one of the mechanisms involved in the etiopathogenesis of RA and thus in the etiology and perpetuation of the disorder [6]. For example, C1 and C3b staining was found to be negative in normal articular cartilage, whereas they were positive in degenerating cartilage biopsies from patients with RA [7]. This strongly suggests the involvement of the classical pathway in the pathogenesis of RA. Similarly, other studies have shown that several individual components of the C system, like C2, C3, C4, C5, and C3d or C4d, are expressed in the synovial fluid of patients with RA [8,9,10]. Furthermore, activation products of the C system may experience a decrease during treatment with anti-tumor necrosis factor-infliximab [11].

The treatment of RA is defined according to agreements by international societies and is based on the use of nonsteroidal anti-inflammatory drugs, glucocorticoids, and primarily disease-modifying antirheumatic drugs (DMARDs), which have been classified as conventional synthetic (csDMARDs), biologic (bDMARDs), and targeted synthetic (tsDMARDs) [12]. However, due to RA’s complexity, which is based on an incompletely elucidated pathophysiological mechanism, further research is needed before RA can become a curable pathology. In this regard, numerous new therapeutic targets are being researched, and potential therapeutic agents are in various stages of testing to achieve complete remission of RA. This includes molecular metabolite targets (such as prostaglandins, thromboxane A2, leukotriene B4 receptor, platelet-activating factor, cannabinoid receptors, inducible nitric oxide), epigenetic targets (DNA methylation, RNA methylation, histone modification), and other protein targets (p38 mitogen-activated protein kinase, complex G protein-coupled receptor kinase 2, granulocyte-macrophage colony-stimulating factor) [13,14].

The role of the C system is worthy of study in RA for several reasons. The C system is a fundamental part of innate immunity, involved in inflammation and tissue destruction in RA. Additionally, the levels and activity of C components can serve as markers for disease activity and prognosis, providing more precise information than other biomarkers. Furthermore, understanding how the C system contributes to RA can lead to the development of specific therapies that modulate this pathway, offering new strategies to treat the disease more effectively. Despite this, there are no studies in the literature that have conducted a comprehensive analysis of the three C pathways in patients with RA. Additionally, the relationship of this complete characterization with disease characteristics, such as disease activity, the presence of ACPA, rheumatoid factor, and systemic inflammation, has not been thoroughly investigated. Therefore, the scope of our study entails a comprehensive analysis of the classical, alternative, and lectin pathways in RA patients. This includes assessing serum levels of individual C components and investigating the relationship between complement activity and RA disease characteristics such as disease activity, rheumatoid factor, and ACPA. We believe this research could lead to better diagnostic markers and therapeutic strategies for RA.

In the present study, we employed advanced next-generation functional assays to evaluate the three C system pathways. Moreover, we quantified various components of the C system associated with all three cascades, encompassing enzymatically generated fragments and serum regulators. Our primary objective was to elucidate the relationships between the functional levels of the three C cascades and specific elements within these pathways in relation to the characteristics of RA, such as disease activity and the presence of the rheumatoid factor or ACPA.

## 2. Results

### 2.1. Demographic and Disease-Related Data

This study included a total of 430 patients diagnosed with RA. Demographic and disease-related characteristics of the participants are shown in Supplementary Table S1. The study population had a mean age of 56 ± 10 years, with 81% of the participants being women. The median duration of the disease was 8 years (interquartile range, IQR, 4–15). At the time of the study, the mean values of C-reactive protein (CRP) and erythrocyte sedimentation rate (ESR) were 2.9 mg/L (IQR 1.4–6.3) and 18 mm/1st hour (IQR 8–34), respectively. Rheumatoid factor was positive in 72% of patients, and 65% were positive for ACPA. The disease activity, as measured by DAS28-ESR (Disease Activity Index in 28 joints), was 3.2 ± 1.4. According to this score, 38% of the patients met the criteria for remission, while 18% and 44% were categorized in the low and moderate/high disease activity groups, respectively. The DAS28-CRP had a value of 2.7 ± 1.1, and the Simple Disease Activity Index (SDAI) and Clinical Disease Activity Index (CDAI) were 13 (IQR 7–20) and 8 (IQR 4–14), respectively. Thirty-six percent of the patients were being treated with prednisone, and 89% were receiving at least one conventional disease-modifying antirheumatic drug (DMARD) in any of its types, methotrexate being the most widely used (75%). Twenty percent of the patients were receiving anti-tumor necrosis factor therapies. The frequency of usage of other treatments and historical disease-related data can be found in Supplementary Table S1.

Functional C assays of the classical, alternative, and lectin pathways; single C components C1q, C1-inhibitor, C2, C4, C4b, C3, C3a, C5, C5a, and C9; and factor D and I, properdin and lectin serum values are shown in Supplementary Table S2. Furthermore, a visual representation of the frequency distribution and violin box plots of the three complement pathways’ functional assays is also provided in Supplementary Figure S2. As depicted in this figure, the classical and alternative pathway functional tests exhibited a normal distribution, while the lectin pathway was skewed to the left, indicating a tendency towards lower values.

### 2.2. Complement System and Disease Activity

**Figure 1:**
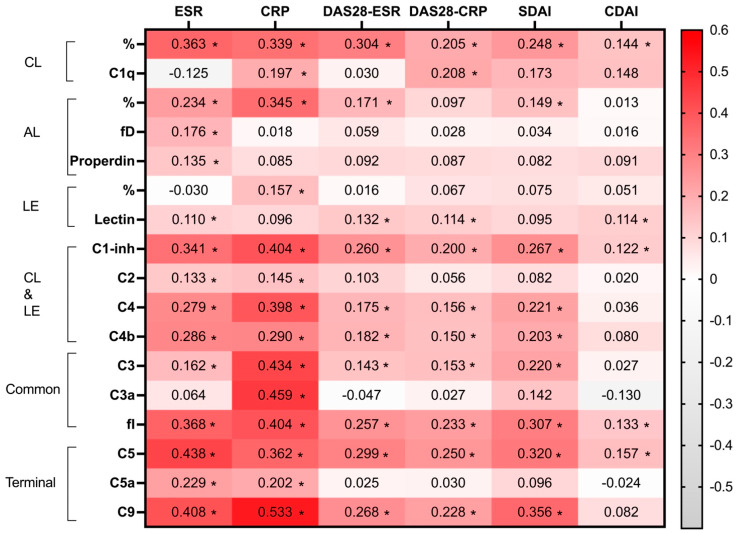
Spearman’s Rho correlation analysis of complement (C) system pathways and individual particles to acute phase reactants and disease activity scores. DAS28: Disease Activity Score in 28 joints, CDAI: Clinical Disease Activity Index, SDAI: Simple Disease Activity Index, CRP: C-reactive protein, ESR: erythrocyte sedimentation rate, CL: classical, LE: lectin: alternative, fI: factor I, fD: factor D. Significant correlation coefficients *p* < 0.05 are depicted as *.

The heatmap presented in Figure 1 illustrates the correlation between functional tests and individual components of the C system with acute phase reactants and composite indices of clinical disease activity. It should be noted that CDAI does not have CRP or ESR in its calculation formula. The noteworthy observation was that almost all correlations were positive, indicating a general trend of positive associations among the variables examined (depicted in red on the heatmap). Notably, CRP exhibited positive and significant correlations with functional tests of all three complement pathways and nearly all individual components (with exceptions for factor D, properdin, and lectin). These correlations were particularly pronounced for the final components of the complement pathway, such as the common and terminal pathways. Similar patterns were observed for ESR. Specifically, ESR showed significant and positive correlations with the classical and alternative pathways (except for lectin) and all complement elements except C1q and C3a (Figure 1).

Disease activity indices, which are calculated based on factors such as acute phase reactants and the presence of swelling or painful joints, exhibited positive and significant relationships with several functional pathway tests and individual C elements. Specifically, all four disease activity indices showed significant and positive associations with the functional test of the classical pathway. While positive relationships were observed for the alternative and lectin pathways, they were less consistent. Notably, CDAI, which does not incorporate CRP in its formula, demonstrated positive and significant relationships with the classical and lectin cascades but not with the alternative pathway. CDAI also revealed positive correlations with the functional tests of the classical and lectin pathways as well as with factor I and C5. Remarkably, correlations of disease activity scores to activated C proteins like C3a and C5a were not significant.

Furthermore, multivariable differences in the complement system between groups of patients in remission or low activity and those with moderate or high activity were analyzed (Table 1 and Table S3). Table 1 displays the analysis for the DAS28-CRP (which is highly influenced by CRP) and CDAI (containing only clinical data without acute phase reactants). This analysis was adjusted for age, sex, and the presence of rheumatoid factor or ACPA. Following adjustment, the classical pathway exhibited higher values (indicating less consumption) in patients with high or moderate activity compared to those in remission or with low activity for both the DAS28-CRP and CDAI scores. Likewise, for the components of the C system in the common and terminal pathways, higher values were observed in the moderate to high disease activity group compared to those in remission or with low activity. However, these differences were more consistently observed for the DAS28-CRP score than for the CDAI score.

### 2.3. Complement System and the Presence of Rheumatoid Factor and Anti-Citrullinated Protein Antibodies

**Figure 2:**
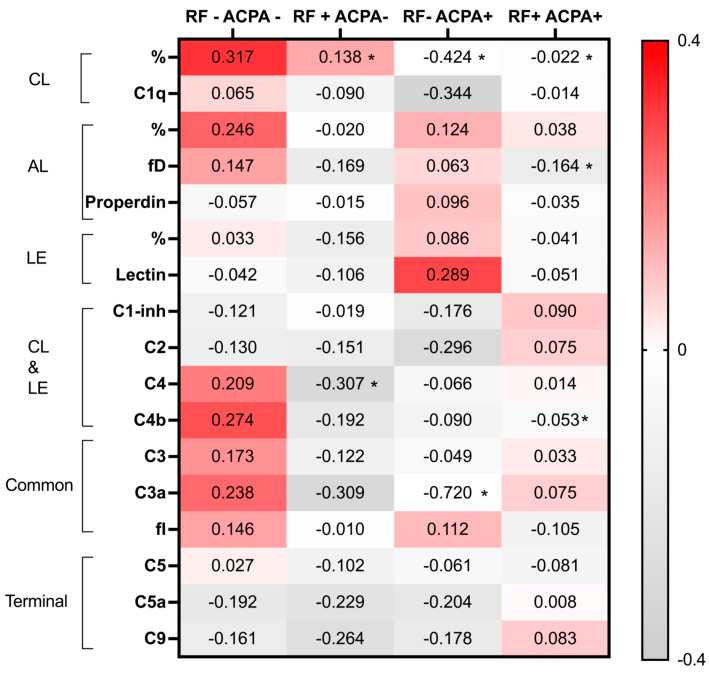
Complement (C) pathways and individual elements values for four categories defined by the presence or absence of rheumatoid factor (RF) and anti-citrullinated protein antibodies (ACPA). Values are shown standardized to allow comparison between them. * Denotes if the comparison between each category, using RF-ACPA as the reference, is significant (*p* < 0.05). CL: classical, LE: lectin: alternative, fI: factor I, fD: factor D.

The differences in the C pathways based on the presence of rheumatoid factor and ACPA are detailed in Figure 2 and Table 2. To analyze this, four groups were established according to the combinations of being positive or negative for rheumatoid factor and ACPA. It is noteworthy that patients with rheumatoid factor and ACPA, or positive for either one but not the other, exhibited lower levels (indicating more consumption) of the classical pathway functional test. This phenomenon was not observed for the alternative and lectin complement pathways. Concerning the individual components of the complement system, patients positive for both rheumatoid factor and ACPA showed lower levels of factor D and C4b, with a tendency to display higher values of C5a and C9 (Table 2).

Furthermore, the standardized values of the C pathways and components are presented in the heatmap of Figure 2. This enables a visual comparison of their values. In general, it is observed that the values of C pathways and components are lower, with some exceptions, as we move towards the right, corresponding to patients with positivity for rheumatoid factor and/or ACPA.

Additionally, the relation of the C system to the use of methotrexate and anti-TNF-alpha therapies is disclosed in Supplementary Table S4. Patients using methotrexate had higher levels of the lectin route, factor D, lectin, and C5a, after multivariable adjustment, compared to those without methotrexate. However, patients under anti-TNF-alpha drugs had lower values for C1-inh, C4, C4b, C3, and C3a compared to those not taking these drugs.

## 3. Discussion

Our study is the first in the literature to comprehensively assess the three pathways of the C system in patients with RA. Notably, this evaluation employed functional tests of the C system, coupled with measurements of individual proteins both upstream and downstream of the three pathways. Based on our findings, disease activity predominantly leads to the upregulation of C elements associated with the terminal pathway. Moreover, a robust correlation exists between the C system and CRP, which was particularly evident for terminal C products. Specifically, disease activity and acute phase reactants are predominantly associated with the classical pathway, as opposed to the other pathways. This association is characterized by higher disease activity corresponding to higher functional test results, indicating reduced consumption. Conversely, the presence of the rheumatoid factor and ACPA is linked to lower values of C3a and C4b as well as reduced functional test results for the classical pathway.

In our study, the classical and alternative pathways displayed a normal distribution pattern. However, the lectin pathway exhibited a left-skewed distribution, indicating a deficiency of this route within our RA population. Lectin pathway deficiency is a prevalent condition, affecting approximately 5–30% of the general population, highlighting the redundancy of the immune system [15]. As a result, it is anticipated that a considerable number of patients with RA would also exhibit this deficiency. Furthermore, although there is limited evidence regarding the significance of the lectin pathway in the pathogenesis of RA, lectin deficiency has been implicated in contributing to the severity of RA and an increased risk of erosive disease, indicating a potentially poorer prognosis [16,17].

CRP, known as an acute-phase serum protein and a mediator of innate immunity, binds to microbial polysaccharides and ligands exposed on damaged cells. Subsequently, CRP initiates the classical pathway by activating C1q [18]. Our analysis aligns with these established functions. We identified a positive correlation between CRP and C1q. Moreover, CRP exhibited a strong correlation with both the classical and alternative pathways but a comparatively weaker correlation with the lectin cascade. The positive correlation of CRP with the classical cascade suggests that higher CRP levels are associated with an upregulation of this cascade. This correlation explains the elevated serum levels of individual C products within the classical pathway and the overall complement system. This association was further supported by the significant and positive association observed between CRP and all analyzed pathways and complement elements, except for factor D, properdin, and lectin. These exceptions may be supported by the fact that factor D and properdin are synthesized in adipose tissue [19] and leucocytes [20], respectively, rather than the liver and therefore may not act as an acute phase reactant. The increased levels of the classical pathway, indicating less consumption, is intriguing. We think that the activation of the classical pathway, whether by CRP or other causes, is outweighed by an excessive production of those C components synthesized in the liver. That is, although the C is consumed during activation, the production of these components can increase as a compensatory response. This compensatory production ensures that, despite the consumption, the measurable levels of C components and functional tests in the blood can be elevated or remain within normal ranges, reflecting the constant activation and ongoing inflammatory response. Furthermore, we cannot rule out that other mechanisms such as genetic variations, differential expression of pathway regulators, or the presence of specific autoantibodies that might selectively influence the classical pathway may also be involved in the findings described in our manuscript. Additionally, as mentioned earlier, most of the patients were deficient to lectin. Notably, the correlation of CRP with complement components increased as we progressed towards those of the terminal pathway. This observation leads to the hypothesis that in patients with RA, CRP may activate the classical pathway. This cascade of events may ultimately lead to an upregulation of the terminal C molecules.

Our study also identified a positive correlation between disease activity and various individual products and functional tests of the C pathways. Importantly, this correlation was notably stronger when disease scores were constructed using CRP or ESR. Specifically, the classical pathway exhibited a positive association with all disease activity scores, including those where acute phase reactants were not considered (e.g., CDAI). Conversely, the alternative pathway showed a less intense relationship with disease activity, and the lectin pathway displayed even weaker associations. Regarding individual C products, disease activity was significantly and positively related to many of them, with a particularly strong association observed for those within the terminal pathway. Notably, the CDAI score, which excludes acute phase reactants, also showed a positive association with higher levels of lectin, C1-inh, factor I, and C5. Furthermore, as mentioned earlier, disease activity demonstrated positive correlations with functional tests. This suggests that higher disease activity corresponds to higher functional test values, indicating reduced consumption of the C pathway. This is supported by the fact that correlations of disease activity scores to activated proteins C, like C3a and C5a, were absent.

In the context of our study, there had been no prior literature examining C activity through functional tests across all three C cascades in RA. In this regard, previous studies, albeit limited, primarily focused on evaluating the classical pathway using the CH50 test. These reports generally indicated that serum CH50 levels are typically normal or elevated in RA [21,22]. For instance, a study involving 54 RA patients observed an increase in serum CH50 levels and a decrease in synovial fluid CH50 levels compared to control groups [23]. Our findings, which demonstrate a positive association between disease activity and functional tests, align with these previous reports. However, it is important to note that no comprehensive characterization of the complement system, such as the one presented in our study, has been previously described in the literature.

Several pieces of evidence have linked C activity to disease activity in RA. For instance, C1 staining was negative in normal articular cartilage but positive in cartilage biopsies from RA patients [7]. Additionally, levels of C1q in serum have been shown to correlate with clinical disease activity in RA patients [24], with similar findings observed in mouse models of RA [6]. Previous studies have also demonstrated the presence of C2, C3, C4, and C5 in rheumatoid synovial fluid [8], along with an increase in the levels of C3d, C4d, and membrane attack complex in the synovial fluid of RA patients [25]. However, it is worth noting that these studies did not analyze the three C pathways in serum, nor did they carry out a detailed characterization of individual C products to the extent presented in our work.

The relationship between the C system and the presence of rheumatoid factor and ACPA, as found in our study, differed from that of disease activity. This implies that positivity for both autoantibodies was broadly associated with lower values of both functional tests and products of the C pathways. In this sense, ACPA antibodies have been described to activate both the classical and alternative pathways of the C system [26]. Furthermore, the rheumatoid factor has been shown to amplify C activation mediated by ACPA [27]. We believe that ACPA and rheumatoid factor likely participate in triggering inflammation-promoting activation of C cascades occurring in RA joints. Therefore, this subset of patients may exhibit lower levels and consumption of C components.

In our study, we found that certain C values were elevated in patients taking methotrexate compared to those not taking it. Conversely, subjects on anti-TNF alpha therapies showed lower levels of some C components compared to those not using these drugs. Given the cross-sectional nature of the study, it is difficult to infer a clear direction in these associations since these therapies may have been used in patients with higher disease activity. Our findings are consistent with previous reports demonstrating that anti-TNF treatments reduce the levels of specific proteins in the complement system [28,29].

Systemic lupus erythematosus (SLE) is a chronic and multisystem immune-mediated disorder characterized by hypocomplementemia, a typical laboratory finding that often reflects activation of the C system by immune complexes [30]. In SLE, accelerated consumption of C components exceeds synthesis, leading to hypocomplementemia. Consequently, functional C tests like CH50 in SLE patients typically reveal low values [31]. These C values often correlate with more severe disease manifestations, particularly renal involvement, and with antibodies to double-stranded DNA. The return of C levels to normal with treatment is considered a positive prognostic sign [32]. However, this pattern differs in RA patients. According to our results, disease activity in RA is associated with increased production, rather than consumption, of various C elements. This highlights the different physiological roles of the C system in these two diseases.

Several therapies targeting the C system have been developed in recent years. For instance, eculizumab and ravulizumab are humanized monoclonal antibodies that bind to C5, blocking its cleavage and the production of terminal C components C5a and the membrane attack complex. Both have been approved for use in hemolytic uremic syndrome and paroxysmal nocturnal hemoglobinuria. Moderate inhibition of C activation has shown significant therapeutic effects in experimental arthritis in mice [33]. However, in a double-blind, placebo-controlled study using eculizumab in twenty-one patients with active RA, C5 blockade did not result in reduced synovial inflammation in RA patients [34]. This does not negate the relevance that the C system may have in RA pathophysiology. In our work, the C system is closely related to disease activity and several features of RA; thus, it could be plausible to aim for a level of C inhibition in RA that offers therapeutic benefits while allowing a certain degree of C activation to mitigate potential adverse effects, such as the risk of infections. This approach could contribute to the development of more targeted and effective therapies for RA [35].

We acknowledge several limitations in our study. Firstly, the assessment of the C system was performed in sera and not in synovial tissue or synovial fluid. In this regard, the presence of split components of the C system on the cartilage surface and in the synovium of RA patients, indicating local deposition, may differ from the expression of C in sera. Therefore, our findings in sera cannot be extrapolated to what may occur in the synovial tissue. Secondly, our study’s cross-sectional design limits our ability to infer causality. Prospective experimental studies targeting specific mechanisms of RA are warranted to analyze the relationship between the complement system and the characteristics of RA. Additionally, the C system is a complex network of a large number of molecules and regulators, making it challenging to provide a complete static picture. Furthermore, we did not recruit controls in our study. However, our intention was not to compare the C system between patients and controls but to study its relationship with disease activity and the presence of rheumatoid factor and ACPA within the patient population. Besides, the exact pathophysiological mechanisms behind our findings cannot be precisely determined. We demonstrate that the C system is related, in different ways, to various aspects of the disease. This remains true even after adjusting for other variables and confounders. Our findings may lay the foundation for future research, potentially of a biological or basic nature, that will provide further insights into this relationship. Moreover, in our study in some cases, the R2 values were not high. However, a heatmap is not intended to demonstrate statistical significance but to evaluate associations by providing a snapshot of how certain variables relate to each other. This visual representation allows us to observe patterns and relationships in the data, even if these patterns do not meet traditional thresholds of statistical significance. Besides, by standardizing the variables, we can better compare the strength of these associations and gain insights into which variables are more closely related to a particular outcome. For this reason, the fact that the correlations were sometimes not significant should not be interpreted as a limitation since the intention of a heatmap is to visually describe a pattern of relationships.

## 4. Conclusions

The fundamental conclusions of our work can be summarized as follows: (1) Lectin pathway values are low in RA patients, indicating a genetic deficiency in this pathway that also exists in the general population. (2) CRP and ESR show a positive correlation with all three C system pathways, with the relationship being more pronounced with common and terminal complement products. (3) Disease activity measured through scores that do not include acute phase reactants showed a positive relationship, after multivariable adjustment, with the classical C cascade but not with the other routes. Additionally, it showed a positive relationship with terminal complement products such as C5. (4) RA patients who tested positive for rheumatoid factor or ACPA antibodies had lower levels of C3a and C4b, indicating consumption of the classical pathway. This contrasts with the overall positive association observed between disease activity and the complement system functional assays and serum protein values.

Our study provides a comprehensive understanding of complement dynamics in RA patients, opening avenues for the development of more precise and effective treatment strategies in the future.

## 5. Materials and Methods

### 5.1. Study Participants

This cross-sectional study included 430 patients with RA who were recruited consecutively. All of them were 18 years old or older and fulfilled the 2010 ACR/EULAR classification criteria [36]. They had been diagnosed by rheumatologists and underwent regular follow-up appointments at rheumatology outpatient clinics. For the purpose of inclusion in the present study, the duration of RA disease was required to be ≥1 year. Since glucocorticoids are often used in the treatment of RA, patients taking prednisone or an equivalent dose ≤ 10 mg/day were allowed to participate in the study. Patients with a history of cancer or any other chronic diseases, including hypothyroidism, heart or respiratory diseases, nephrotic syndrome, as well as those showing evidence of active infection, were excluded from participation in the study. A flowchart illustrating the excluded and included patients is illustrated in Supplementary Figure S1. The study protocol was approved by the Institutional Review Committee at Hospital Universitario de Canarias and at Hospital Universitario Doctor Negrín (both in Spain), and all subjects provided informed written consent (approval no. 2019-452-1). All research activities were conducted in strict adherence to relevant guidelines and regulations and in accordance with the principles outlined in the Declaration of Helsinki.

### 5.2. Data Collection, Laboratory Assessments, and Carotid Ultrasound Evaluation

Participants enrolled in the study underwent a thorough examination, including the completion of a cardiovascular risk factor and medication use questionnaire. A comprehensive physical examination was conducted, encompassing measurements such as body mass index (BMI) calculated as weight in kilograms divided by the square of the height in meters, abdominal circumference, and assessment of systolic and diastolic blood pressure under standardized conditions. Additionally, information regarding smoking, diabetes, and hypertension was gathered. Specific diagnoses and medication details were verified through a review of medical records.

Blood samples were collected from fasting patients (9–12 h) via venipuncture into vacutainer tubes. For serum collection, blood samples were allowed to clot at room temperature for 30 min, followed by centrifugation to separate the serum. The serum samples were then transferred to labeled secondary tubes and stored at 2–8 °C for short-term storage or frozen at −80 °C for long-term storage. All samples were transported to the laboratory on ice to maintain stability. Proper labeling and documentation accompanied each sample to ensure accurate identification and analysis. Cholesterol, triglycerides, and HDL cholesterol were measured using the enzymatic colorimetric assay. LDL cholesterol was calculated using the Friedewald formula (LDL = total cholesterol-HDL-tryglicerides/5). The erythrocyte sedimentation rate (ESR) was determined using the Westergren method. Venous blood samples were collected and mixed with sodium citrate in a 4:1 ratio. The mixture was placed in Westergren tubes and allowed to stand upright at room temperature for one hour. The distance the erythrocytes settled was measured in millimeters per hour (mm/hr). High-sensitivity C-reactive protein (hs-CRP) levels were measured using a high-sensitivity immunoassay. Disease activity in patients with RA was measured using the Disease Activity Score (DAS28) in 28 joints [37], the Clinical Disease Activity Index (CDAI) [38], and the Simple Disease Activity Index (SDAI) [39]. DAS28-ESR and DAS28-CRP were classified into distinct categories based on predefined thresholds: remission (<2.6), low (>2.6 to 3.2), moderate (>3.2 to 5.1), or high disease activity (>5.1) as previously described [40]. Likewise, SDAI categories were defined as follows: remission (<3.3), moderate disease activity (<11), high disease activity (<26), and very high disease activity (>26). Concurrently, the CDAI was categorized into remission (<2.8), moderate disease activity (<10), high disease activity (<22), and very high disease activity (>22). These categorizations adhere to established criteria [41].

### 5.3. Complement Assessments

The SVAR functional C assays under the Wieslab^®^ brand (Sweden) were used to assess classical, alternative, and lectin pathways activity. These tests integrate principles from the hemolytic assay for C function with the utilization of labeled antibodies specifically targeting the neoantigen produced as a result of C activation. Indeed, the amount of neoantigen generated was directly proportional to the functional activity of the C pathways. Microtiter strip wells were coated with classical, alternative, or lectin pathway-specific activators. In this procedure, the patient’s serum underwent dilution with a specific blocker to ensure activation of only the studied C pathway. During the incubation of the diluted patient serum in the wells, the specific coating activated C. Subsequently, the wells were washed, and the presence of C5b-9 was detected using an alkaline phosphatase antibody specifically labeled against the neoantigen expressed during membrane attack complex (MAC) formation. Following an additional washing step, specific antibodies were detected by incubating with an alkaline phosphatase substrate solution. The intensity of the color developed correlated with the amount of C activation and was measured in terms of absorbance (optical density). The quantity of formed membrane attack complex (MAC) neo-epitope reflected the activity of the C cascade. The result was expressed semi-quantitatively by calculating the optical density ratio between a positive control and the sample. It is crucial to note that for the classical, alternative, and lectin cascade values, lower levels indicated a higher activation of the respective pathway. Wieslab^®^ validated these functional assays by studying their correlation and concordance with the classical CH50 and AH50 hemolytic tests (https://www.svarlifescience.com/ accessed 1 June 2024). Additionally, C individual elements were assessed through MILLIPLEX^®^ map Multiplex Detection (MERCK^®^, Cat. No. HCMP1MAG-19K and No. HCMP2MAG-19K). To achieve a comprehensive characterization of all complement pathways, panels were devised to evaluate various components, including C1q (classical pathway); factor D and properdin (alternative pathway); lectin (lectin pathway); C1-inhibitor; C2, C4, and C4b (classical and lectin pathways); C3, C3a, and C4b (common pathway); as well as C5, C5a, and C9 (terminal pathway). Both intra- and inter-coefficients of variability for these assays were maintained below 10%.

### 5.4. Statistical Analysis

Demographic and clinical characteristics in patients with RA were described as mean (standard deviation) or percentages for categorical variables. For non-normally distributed continuous variables, data were expressed as median and interquartile range (IQR). The association between RA features and circulating C system molecules and pathways was evaluated through linear multivariable regression analyses. All analyses were conducted with a 5% two-sided significance level using Stata software, version 17/BE (StataCorp, College Station, TX, USA). *p*-values < 0.05 were considered statistically significant.

## Figures and Tables

**Table 1:** Complement system differences between patients in remission or with low activity compared to moderate or high disease activity.

	DAS28-CRP				CDAI			
	Remission and Low Activity	Moderate and High Activity			Remission and Low Activity	Moderate and High Activity		
	*n* = 295	*n* = 126	*p*	*p* *	*n* = 277	*n* = 146	*p*	*p* *
Functional C assays, %								
Classical pathway	**94 ± 24**	**102 ± 23**	**0.005**	**0.007**	**94 ± 24**	**100 ± 23**	**0.031**	**0.042**
Alternative pathway	90 ± 25	92 ± 28	0.46		91 ± 24	90 ± 28	0.73	
Lectin pathway	49 (7–102)	53 (11–111)	0.55		50 (7–101)	53 (10–111)	0.50	
Individual C components								
Classical pathway								
C1q, mg/dL	32.5 ± 6.9	34.2 ± 10.3	0.29		32.4 ± 6.9	34.4 ± 9.7	0.20	
Alternative pathway								
Factor D, mg/dL	0.17 ± 0.07	0.17 ± 0.07	0.56		0.17 ± 0.07	0.17 ± 0.06	0.96	
Properdin, mg/dL	1.27 ± 0.45	1.32 ± 0.34	0.22		1.27 ± 0.35	1.30 ± 0.35	0.43	
Lectin pathway								
Lectin, mg/dL	0.07 (0.03–0.18)	0.09 (0.03–0.23)	0.12		0.07 (0.03–0.18)	0.09 (0.04–0.22)	0.23	
Classical and lectin pathways								
C1-inhibitor, mg/dL	**31.8 ± 6.4**	**34.0 ± 7.8**	**0.003**	**0.003**	**31.9 ± 6.5**	**33.5 ± 7.7**	**0.037**	**0.030**
C2, mg/dL	6.4 (3.9–10.5)	6.9 (4.2–10.7)	0.65		6.5 (4.0–10.5)	6.5 (4.0–10.7)	0.91	
C4, mg/dL	**26.6 ± 10.0**	**28.9 ± 10.6**	**0.032**	**0.012**	27.0 ± 9.9	28.0 ± 10.8	0.37	
C4b, mg/dL	**5.9 ± 3.2**	**6.9 ± 3.4**	**0.008**	**0.002**	6.0 ± 3.2	6.6 ± 3.4	0.11	0.065
Common pathway								
C3, mg/dL	**139 ± 28**	**146 ± 30**	**0.027**	**0.031**	141 ± 28	141 ± 31	0.89	
C3a, mg/dL	34.4 ± 9.5	33.5 ± 9.8	0.68		34.1 ± 9.4	34.3 ± 10.1	0.90	
Factor I, mg/dL	**3.9 ± 1.1**	**4.5 ± 1.2**	**<0.001**	**<0.001**	**4.0 ± 1.1**	**4.3 ± 1.2**	**0.017**	**0.007**
Terminal pathway								
C5, mg/dL	**3.6 ± 1.4**	**4.5 ± 2.5**	**<0.001**	**<0.001**	**3.7 ± 1.5**	**4.2 ± 2.3**	**0.017**	**0.008**
C5a, mg/dL	1.2 ± 1.0	1.1 ± 0.6	0.54		1.2 ± 1.0	1.1 ± 0.6	0.10	0.16
C9, mg/dL	**0.9 ± 0.5**	**1.1 ± 0.6**	**<0.001**	**<0.001**	1.0 ± 0.5	1.0 ± 0.6	0.12	0.076

* Adjusted for age, sex, and positivity for rheumatoid factor or anti-citrullinated protein antibodies. CRP: C-reactive protein; DAS28: Disease Activity Score in 28 joints; CDAI: Clinical Disease Activity Index. Significant *p* values are depicted in bold.

**Table 2:** Association of complement system pathways and individual elements with the presence of rheumatoid factor and anti-citrullinated protein antibodies.

	RF − ACPA−	RF + ACPA−		RF − ACPA+		RF + ACPA+	
	*n* = 86	*n* = 48		*n* = 24		*n* = 229	
Functional complement assays, %			*p*		*p*		*p*
Classical pathway	103 ± 18	99 ± 28	0.32	**85 ± 29**	**0.002**	**95 ± 24**	**0.014**
Alternative pathway	97 ± 20	89 ± 29	0.15	93 ± 25	0.62	91 ± 27	0.12
Lectin pathway	56 (8–107)	38 (4–90)	0.34	48 (19–96)	0.83	47 (7–106)	0.59
Individual complement components							
Classical pathway							
C1q, mg/dL	33.5 ± 9.1	32.2 ± 4.3	0.64	30.3 ± 6.3	0.33	33.0 ± 7.8	0.84
Alternative pathway							
Factor D, mg/dL	0.2 ± 0.08	0.02 ± 0.05	0.075	0.02 ± 0.07	0.68	**0.02 ± 0.07**	**0.012**
Properdin, mg/dL	1.3 ± 0.3	1.3 ± 0.3	0.83	1.3 ± 0.4	0.57	1.3 ± 0.4	0.87
Lectin pathway							
Lectin, mg/dL	0.08 (0.03–0.18)	0.07 (0.03–0.17)	0.77	0.12 (0.04–0.33)	0.22	0.08 (0.03–0.19)	0.95
Classical and lectin pathways							
C1-inhibitor, mg/dL	31.6 ± 6.7	32.2 ± 8.0	0.63	31.2 ± 6.8	0.81	32.5 ± 6.9	0.095
C2, mg/dL	5.6 (4.1–9.5)	7.0 (3.7–10.1)	0.80	4.9 (4.2–10.7)	0.50	7.3 (3.9–11.5)	0.14
C4, mg/dL	**29.6 ± 11.2**	**24.3 ± 8.8**	**0.005**	26.7 ± 9.8	0.25	27.6 ± 10.4	0.13
C4b, mg/dl	7.1 ± 3.8	5.6 ± 3.1	0.20	5.9 ± 3.1	0.17	**6.0 ± 3.4**	**0.016**
Common pathway							
C3, mg/dL	146 ± 29	138 ± 27	0.11	139 ± 23	0.36	142 ± 28	0.30
C3a, mg/dL	36.4 ± 8.9	31.2 ± 9.5	0.099	**27.3 ± 6.0**	**0.021**	34.8 ± 9.5	0.51
Factor I, mg/dL	4.3 ± 1.4	4.1 ± 1.0	0.42	4.2 ± 1.6	0.94	4.0 ± 1.2	0.071
Terminal pathway							
C5, mg/dL	4.0 ± 1.8	3.7 ± 1.4	0.49	3.9 ± 2.3	0.92	3.9 ± 2.0	0.59
C5a, mg/dL	1.0 ± 0.6	1.0 ± 0.4	0.85	1.0 ± 0.4	0.96	1.2 ± 1.1	0.058
C9, mg/dL	0.9 ± 0.4	0.8 ± 0.4	0.58	0.9 ± 0.4	0.95	1.0 ± 0.6	0.051

*p* values represent differences between categories assuming RF − ACPA− as the reference category. RF: rheumatoid factor, ACPA: anti-citrullinated protein antibodies. Significant *p* values are depicted in bold.

## Data Availability

Data will be made available on request.

## Supplementary Materials

The following supporting information can be downloaded at: https://www.mdpi.com/article/10.3390/ijms25158360/s1.
