# Supplementary material for: Complete Description of the Three Pathways of the Complement System in a Series of 430 Patients with Rheumatoid Arthritis
Source: Int J Mol Sci. 2024 Jul 31;25(15):8360. doi: 10.3390/ijms25158360 (PMC11312865; doi:10.3390/ijms25158360)
Supplement: Supplementary file 1 [file ijms-25-08360-s001.zip › ijms-3109491-supplementary.pdf]

Supplementary Table S1. Demographics and disease related data in RA patients.

	Rheumatoid arthritis (n=430)
Age, years	55 ± 10
Female, n (%)	350 (81)
BMI, kg/m2	28 ± 5
Abdominal circumference, cm	97 ± 13
Hip circumference, cm	106 ± 11
Abdominal to hip ratio	0.92 ± 0.08
Cardiovascular risk factors, n (%)	
Current smoker	93 (22)
Obesity	137 (32)
Hypertension	148 (34)
Diabetes Mellitus	54 (13)
Statins	139 (32)
Disease related data	
Disease duration, years	8 (4-15)
CRP at time of study, mg/l	2.7 (1.3-6.1)
ESR at time of study, mm/1st hour	18 (7-32)
Rheumatoid factor, n (%)	303 (72)
ACPA, n (%)	253 (65)
DAS28-ESR	3.13 ± 1.35
Remission, n (%)	166 (40)
Low activity, n (%)	76 (18)
Moderate activity, n (%)	138 (33)
High activity, n (%)	39 (9)
DAS28-PCR	2.73 ± 1.08
Remission, n (%)	224 (53)
Low activity, n (%)	71 (17)
Moderate activity, n (%)	111 (26)
High activity, n (%)	15 (4)
SDAI	12 (7-19)
Remission, n (%)	33 (8)
Low activity, n (%)	155 (37)
Moderate activity, n (%)	179 (43)
High activity, n (%)	52 (12)
CDAI	8 (4-14)
Remission, n (%)	79 (19)
Low activity, n (%)	198 (47)
Moderate activity, n (%)	116 (27)
High activity, n (%)	30 (7)
History of extraarticular manifestations, n (%)	38 (10)
Erosions, n (%)	166 (43)
Current drugs, n (%)	
Prednisone	156 (36)
Prednisone doses, mg/day	5 (3-5)
NSAIDs	194 (45)
DMARDs	373 (87)
Methotrexate	316 (73)
Leflunomide	94 (22)
Hydroxychloroquine	45 (18)
Salazopyrin	28 (7)

Anti TNF therapy	83 (19)
Tocilizumab	23 (5)
Rituximab	7 (2)
Abatacept	12 (3)
JAK inhibitors	20 (5)
Baricitinib	6 (1)
Tofacitinib	11 (3)

Data represent mean ± SD or median (IQR) when data were not normally distributed. CRP: C reactive protein; ACPA: Anti-citrullinated protein antibodies. NSAID: Nonsteroidal anti-inflammatory drugs; DMARD: disease-modifying antirheumatic drug. TNF: tumor necrosis factor; Obesity; ESR: erythrocyte sedimentation rate. BMI: body mass index; DAS28: Disease Activity Score in 28 joints. CDAI: Clinical Disease Activity Index; SDAI: Simple Disease Activity Index.

Supplementary Table S2. Functional test and individual elements values of the complement system in RA patients.

Functional complement assays, %	
Classical pathway	96 ± 24
Alternative pathway	90 ± 26
Lectin pathway	50 (7-106)
Individual complement components	
Classical pathway	
C1q, mg/dl	33 ± 8
Alternative pathway	
Factor D, mg/dl	0.17 ± 0.07
Properdin, mg/dl	1.3 ± 0.4
Lectin pathway	
Lectin, mg/dl	0.08 (0.03-0.19)
Classical and lectin pathways	
C1q inhibitor, mg/dl	32 ± 7
C2, mg/dl	7 (4-11)
C4, mg/dl	27 ± 10
C4b, mg/dl	6 ± 3
Common pathway	
C3, mg/dl	141 ± 29
C3a, mg/dl	34 ± 10
Factor I, mg/dl	4 ± 1
Terminal pathway	
C5, mg/dl	3.9 ± 1.9
C5a, mg/dl	1.0 (0.7-1.4)
C9, mg/dl	1.0 (0.6-1.3)

Supplementary Table S3. Complement system differences of patients in remission or with low activity compared to moderate or high disease activity.

	DAS2-ESR				DAS28-CRP			
	Remission and low activity n=242	Moderate and high activity n=177	p	p*	Remission and low activity n=295	Moderate and high activity n=126	p	p*
Functional C assays, %								
Classical pathway	**90 ± 23**	**104 ± 23**	**<0.001**	**<0.001**	**94 ± 24**	**102 ± 23**	**0.005**	**0.007**
Alternative pathway	**87 ± 26**	**96 ± 24**	**<0.001**	**0.003**	90 ± 25	92 ± 28	0.46	
Lectin pathway	49 (8-99)	56 (10-110)	0.40		49 (7-102)	53 (11-111)	0.55	
Individual C components								
Classical pathway								
C1q, mg/dl	32 ± 7	34 ± 10	0.18	0.24	33 ± 7	34 ± 10	0.29	
Alternative pathway								
Factor D, mg/dl	**0.16 ± 0.07**	**0.18 ± 0.07**	**0.091**	**0.010**	0.17 ± 0.07	0.17 ± 0.07	0.56	
Properdin, mg/dl	1.25 ± 0.35	1.32 ± 0.35	0.054	0.050	1.27 ± 0.45	1.32 ± 0.34	0.22	
Lectin pathway								
Lectin, mg/dl	0.07 (0.03-0.17)	0.09 (0.04-0.22)	0.22		0.07 (0.03-0.18)	0.09 (0.03-0.23)	0.12	
Classical and lectin pathways								
C1q inhibitor, mg/dl	**31 ± 6**	**34 ± 7**	**<0.001**	**<0.001**	**32 ± 6**	**34 ± 8**	**0.003**	**0.003**
C2, mg/dl	6 (4-10)	7 (4-11)	0.43		6 (4-11)	7 (4-11)	0.65	
C4, mg/dl	**25 ± 10**	**30 ± 10**	**<0.001**	**<0.001**	**27 ± 10**	**29 ± 11**	**0.032**	**0.012**
C4b, mg/dl	**6 ± 3**	**7 ± 3**	**<0.001**	**<0.001**	**6 ± 3**	**7 ± 3**	**0.008**	**0.002**
Common pathway								
C3, mg/dl	**137 ± 28**	**146 ± 29**	**0.001**	**0.005**	**139 ± 28**	**146 ± 30**	**0.027**	**0.031**
C3a, mg/dl	34 ± 10	35 ± 9	0.74		34 ± 10	34 ± 10	0.68	
Factor I, mg/dl	**3.8 ± 1.1**	**4.4 ± 1.2**	**<0.001**	**<0.001**	**3.9 ± 1.1**	**4.5 ± 1.2**	**<0.001**	**<0.001**
Terminal pathway								
C5, mg/dl	**3.5 ± 1.3**	**4.4 ± 2.2**	**<0.001**	**<0.001**	**3.6 ± 1.4**	**4.5 ± 2.5**	**<0.001**	**<0.001**
C5a, mg/dl	1.1 ± 0.7	1.2 ± 1.1	0.30		1.2 ± 1.0	1.1 ± 0.6	0.54	
C9, mg/dl	**0.85 ± 0.45**	**1.16 ± 0.60**	**<0.001**	**<0.001**	**0.9 ± 0.5**	**1.1 ± 0.6**	**<0.001**	**<0.001**

	SDAI				CDAI			
	Remission and low activity n=188	Moderate and high activity n=231	p	p*	Remission and low activity n=277	Moderate and high activity n=146	p	p*
Functional C assays, %								
Classical pathway	**90 ± 25**	**101 ± 22**	**<0.001**	**<0.001**	**94 ± 24**	**100 ± 23**	**0.031**	**0.042**
Alternative pathway	**86 ± 25**	**94 ± 25**	**0.005**	**0.004**	91 ± 24	90 ± 28	0.73	
Lectin pathway	49 (6-99)	52 (10-110)	0.54		50 (7-101)	53 (10-111)	0.50	

Individual C components								
Classical pathway								
C1q, mg/dl	**31 ± 6**	**34 ± 9**	**0.042**	**0.032**	32 ± 7	34 ± 10	0.20	
Alternative pathway								
Factor D, mg/dl	0.17 ± 0.07	0.17 ± 0.07	0.72		0.17 ± 0.07	0.17 ± 0.06	0.96	
Properdin, mg/dl	1.26 ± 0.35	1.30 ± 0.35	0.27		1.27 ± 0.35	1.30 ± 0.35	0.43	
Lectin pathway								
Lectin, mg/dl	0.07 (0.03-0.18)	0.08 (0.03-0.21)	0.49		0.07 (0.03-0.18)	0.09 (0.04-0.22)	0.23	
Classical and lectin pathways								
C1q inhibitor, mg/dl	**31 ± 6**	**34 ± 8**	**<0.001**	**<0.001**	**32 ± 7**	**33 ± 8**	**0.037**	**0.030**
C2, mg/dl	6 (4-10)	7 (4-11)	0.069	0.053	6 (4-11)	7 (4-11)	0.91	
C4, mg/dl	**25 ± 9**	**29 ± 11**	**<0.001**	**<0.001**	27 ± 10	28 ± 11	0.37	
C4b, mg/dl	**6 ± 3**	**7 ± 3**	**0.002**	**0.002**	6 ± 3	7 ± 3	0.11	0.065
Common pathway								
C3, mg/dl	**135 ± 26**	**146 v 30**	**<0.001**	**<0.001**	141 ± 28	141 ± 31	0.89	
C3a, mg/dl	33 ± 9	36 ± 10	0.11	0.088	34 ± 9	34 ± 10	0.90	
Factor I, mg/dl	**3.8 ± 1.1**	**4.3 ± 1.2**	**<0.001**	**<0.001**	**4.0 ± 1.1**	**4.3 ± 1.2**	**0.017**	**0.007**
Terminal pathway								
C5, mg/dl	**3.4 ± 1.3**	**4.3 ± 2.1**	**<0.001**	**<0.001**	**3.7 ± 1.5**	**4.2 ± 2.3**	**0.017**	**0.008**
C5a, mg/dl	**1.1 ± 0.6**	**1.2 ± 1.1**	**0.062**	**0.026**	1.2 ± 1.0	1.1 ± 0.6	0.10	0.16
C9, mg/dl	**0.8 ± 0.4**	**1.1 ± 0.6**	**<0.001**	**<0.001**	1.0 ± 0.5	1.0 ± 0.6	0.12	0.076

*Adjusted for age, sex, and positivity for rheumatoid factor or anti-citrullinated protein antibodies. DAS28: Disease Activity Score in 28 joints; CDAI: Clinical Disease Activity Index; SDAI: Simple Disease Activity Index. CRP: C reactive protein; ESR: erythrocyte sedimentation rate.

Supplementary Table S4. Complement system differences of patients with and without methotrexate and aniTNF-alpha

	Methotrexate				antiTNF-alpha			
	No=114	Yes=316	p	p*	No=347	Yes=83	p	p*
Functional C assays, %								
Classical pathway	95 ± 27	96 ± 23	0.71		**97 ± 24**	**90 ± 23**	**0.029**	0.42
Alternative pathway	93 ± 28	89 ± 25	0.27		**92 ± 26**	**83 ± 22**	**0.005**	0.31
Lectin pathway	**35 (4-85)**	**58 (9-109)**	**0.021**	**0.031**	48 (5-106)	63 (20-107)	0.17	0.41
Individual C components								
Classical pathway								
C1q, mg/dl	32 ± 7	33 ± 8	0.66		33 ± 8	33 ± 7	0.92	
Alternative pathway								
Factor D, mg/dl	**0.15 ± 0.06**	**0.18 ± 0.07**	**0.001**	**0.021**	**0.17 ± 0.07**	**0.19 ± 0.07**	**0.021**	0.16
Properdin, mg/dl	1.23 ± 0.36	1.31 ± 0.35	0.061	0.12	1.28 ± 0.35	1.32 ± 0.36	0.34	
Lectin pathway								
Lectin, mg/dl	**0.06 (0.03-0.13)**	**0.08 (0.03-0.21)**	**0.014**	**0.031**	0.07 (0.03-0.19)	0.11 (0.05-0.20)	0.14	0.27
Classical and lectin pathways								
C1 inhibitor, mg/dl	32 ± 7	33 ± 7	0.70		**33 ± 7**	**29 ± 6**	**<0.001**	**<0.001**
C2, mg/dl	6.0 (3.6-9.3)	7.1 (4.1-10.9)	0.13	0.13	6.4 (3.8-10.7)	8.0 (5.1-10.5)	0.31	
C4, mg/dl	27 ± 11	28 ± 10	0.54		**28 ± 11**	**24 ± 8**	**<0.001**	**<0.001**
C4b, mg/dl	5.9 ± 3.4	6.3 ± 3.2	0.23		**6.4 ± 3.4**	**5.4 ± 2.8**	**0.024**	**0.010**
Common pathway								
C3, mg/dl	145 ± 32	139 ± 28	0.054	0.18	**144 ± 29**	**129 ± 25**	**<0.001**	**0.002**
C3a, mg/dl	33 ± 10	35 ± 9	0.31		**35 ± 10**	**28 ± 6**	**0.003**	**0.003**
Factor I, mg/dl	3.9 ± 1.2	4.1 ± 1.2	0.12	0.21	4.1 ± 1.2	4.0 ± 1.1	0.73	
Terminal pathway								
C5, mg/dl	3.6 ± 1.5	4.0 ± 2.0	0.064	0.16	4.0 ± 2.0	3.8 ± 1.3	0.58	
C5a, mg/dl	**0.9 ± 0.6**	**1.2 ± 1.0**	**0.004**	**0.041**	**1.1 ± 0.9**	**1.4 ± 0.7**	**0.018**	0.20
C9, mg/dl	**0.9 ± 0.5**	**1.0 ± 0.6**	**0.045**	0.12	1.0 ± 0.5	1.0 ± 0.6	0.71	

*Adjusted for age, sex, and positivity for rheumatoid factor or anti-citrullinated protein antibodies.
TNF: Tumor necrosis factor

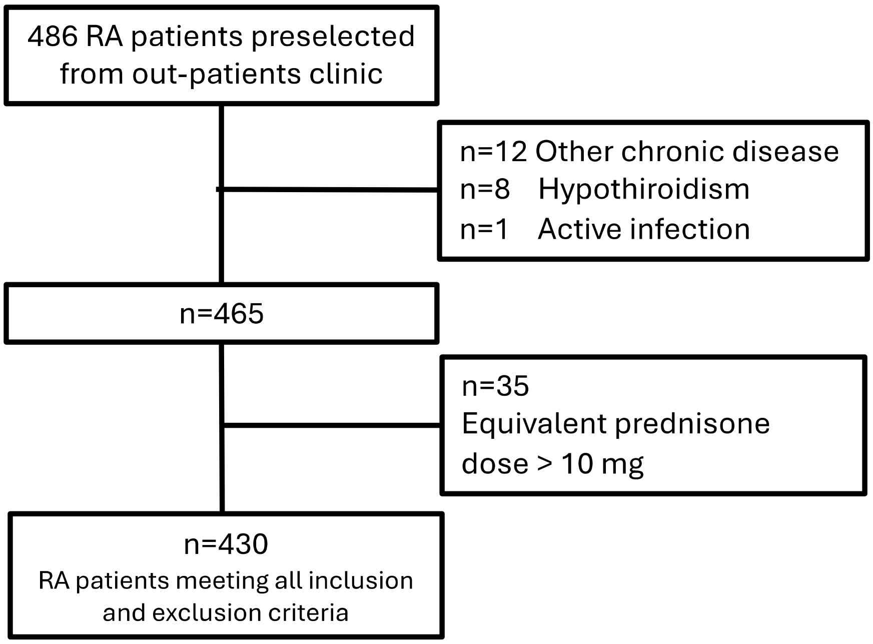

Supplementary Figure S1. Flowchart illustrating the excluded and included patients.

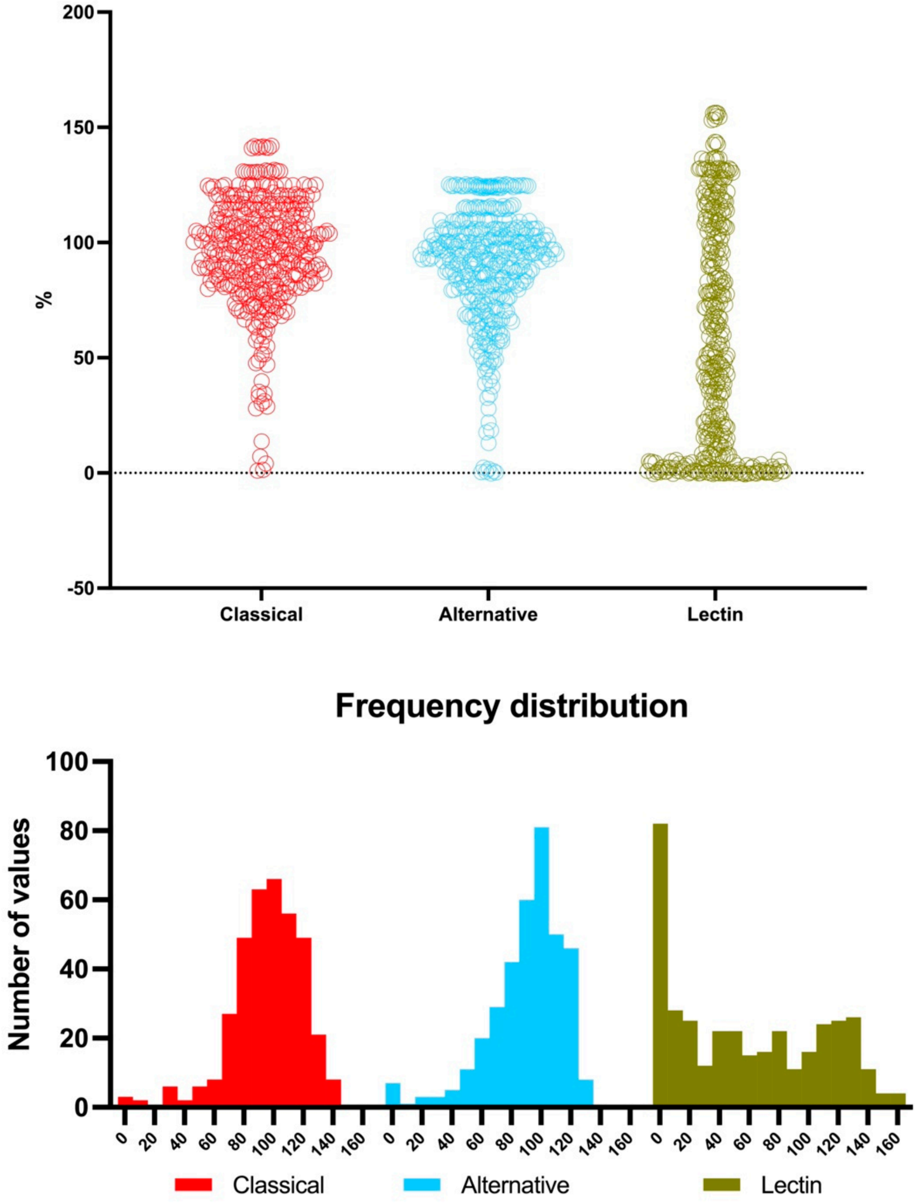

Supplementary Figure S2. Violin plots and histograms of complement system three pathways.
